# Dual Role of the P2X7 Receptor in Dendritic Outgrowth during Physiological and Pathological Brain Development

**DOI:** 10.1523/JNEUROSCI.0805-22.2022

**Published:** 2023-02-15

**Authors:** Paula Mut-Arbona, Lumei Huang, Mária Baranyi, Pál Tod, András Iring, Francesco Calzaferri, Cristobal de los Ríos, Beáta Sperlágh

**Affiliations:** ^1^Laboratory of Molecular Pharmacology, Institute of Experimental Medicine, 1083, Budapest, Hungary; ^2^János Szentágothai Doctoral School, Semmelweis University, 1085, Budapest, Hungary; ^3^Instituto-Fundación Teófilo Hernando and Departamento de Farmacología, Facultad de Medicina, Universidad Autónoma de Madrid, 28049, Madrid, Spain

**Keywords:** ATP, behavior, dendrites, P2X7, schizophrenia, Sholl analysis

## Abstract

At high levels, extracellular ATP operates as a “danger” molecule under pathologic conditions through purinergic receptors, including the ionotropic P2X7 receptor (P2X7R). Its endogenous activation is associated with neurodevelopmental disorders; however, its function during early embryonic stages remains largely unclear. Our objective was to determine the role of P2X7R in the regulation of neuronal outgrowth. For this purpose, we performed Sholl analysis of dendritic branches on primary hippocampal neurons and in acute hippocampal slices from WT mice and mice with genetic deficiency or pharmacological blockade of P2X7R. Because abnormal dendritic branching is a hallmark of certain neurodevelopmental disorders, such as schizophrenia, a model of maternal immune activation (MIA)-induced schizophrenia, was used for further morphologic investigations. Subsequently, we studied MIA-induced behavioral deficits in young adult mice females and males. Genetic deficiency or pharmacological blockade of P2X7R led to branching deficits under physiological conditions. Moreover, pathologic activation of the receptor led to deficits in dendritic outgrowth on primary neurons from WT mice but not those from P2X7R KO mice exposed to MIA. Likewise, only MIA-exposed WT mice displayed schizophrenia-like behavioral and cognitive deficits. Therefore, we conclude that P2X7R has different roles in the development of hippocampal dendritic arborization under physiological and pathologic conditions.

**SIGNIFICANCE STATEMENT** Our main finding is a novel role for P2X7R in neuronal branching in the early stages of development under physiological conditions. We show how a decrease in the expression of P2X7R during brain development causes the receptor to play pathologic roles in adulthood. Moreover, we studied a neurodevelopmental model of schizophrenia and found that, at higher ATP concentrations, endogenous activation of P2X7R is necessary and sufficient for the development of positive and cognitive symptoms.

## Introduction

Extracellular ATP is an important signaling molecule that regulates several cellular functions and activates purinergic P2 receptors, which are expressed in the early embryonic stage during brain development, thus influencing cellular differentiation, proliferation, and apoptosis (Burnstock and Ulrich, 2011; Cavaliere et al., 2015). The ionotropic receptors P2X and particularly the P2X7 receptor (P2X7R) have received considerable attention in the past decade because of their involvement in diseases of the CNS (Sperlágh and Illes, 2014), including psychiatric disorders (Andrejew et al., 2020).

P2X7R is sensitive to a high concentration of ATP, which has been recognized as a “danger signal” (Rodrigues et al., 2015; Cicko et al., 2018), suggesting a specific role for the receptor under pathologic conditions when cell loss or inflammation results in an ATP-rich extracellular milieu. Nevertheless, a wealth of data indicates that P2X7R is also involved in the regulation of physiological functions, such as neurotransmitter release (Sperlágh et al., 2002), memory (Campos et al., 2014), and cognition (Zheng et al., 2017) as well as the development of the nervous system. Extracellular ATP is closely associated with the development of neuritic processes on cultured hippocampal neurons mediated by a tissue-nonspecific alkaline phosphatase, which is functionally related to P2X7R. Both ATP and P2X7R are important for axonal development and exert different regulatory effects *in vitro* (Díaz-Hernandez et al., 2008; Díez-Zaera et al., 2011). However, it is unclear how their presence and absence impact the *in vitro* development and morphology of dendrites on primary pyramidal neurons isolated from the hippocampus.

Disruption of dendrite morphology is associated with various neurologic disorders related to intellectual disabilities and cognitive deficits, such as autism spectrum disorder (ASD), Rett syndrome, and Down syndrome (Moser, 1999). Neurons in some regions of the brain affected in schizophrenia show reduced dendritic length (Kalus et al., 2000), and other alterations in dendritic morphology have been observed on pyramidal cells (Moyer et al., 2015; Shelton et al., 2015), suggesting that dendritic outgrowth is abnormal in schizophrenic patients (Grubisha et al., 2021).

Schizophrenia is a common psychiatric disorder, that affects 1% of the worldwide population (McGrath et al., 2008). Schizophrenia is regarded as a neurodevelopmental psychiatric disorder, especially a dynamic disorder, in which changes in brain development and maturation might be because of the interaction of various genetic, epigenetic, and environmental factors (Patterson, 2009; Brown, 2011). Insults in prenatal or early life may act predispose individuals to nervous system dysfunction, resulting in morphologic and functional alterations in individual neurons and their networks. These early-life first-hit events could include severe maternal infections or autoimmune disorders, which are risk factors for schizophrenia (Müller et al., 2015). Several epidemiological studies have indicated an association between maternal bacterial and viral infections (e.g., maternal influenza infection) during pregnancy and an increased incidence of schizophrenia in the offspring after puberty (Shi et al., 2003; Meyer et al., 2005; Solek et al., 2018). A recent meta-analysis showed that the expression of genes linked to schizophrenia and other neurodevelopmental diseases is dysregulated in maternal immune activation (MIA) mouse models (Laighneach et al., 2021). Recently, the potential of P2X7Rs as treatment targets for schizophrenia was examined. Indeed, in a phencyclidine-induced schizophrenia mouse model, both genetic deletion of P2X7R and pharmacological inhibition of P2X7R attenuated schizophrenia-like behavior (Koványi et al., 2016).

The objective of the present study was to assess the regulatory effect of P2X7R on neuronal outgrowth and morphology in primary cultures of murine hippocampal neurons and acute hippocampal slices. We also investigated dendritic morphology in a mouse model of schizophrenia. The aim of this research was to study the association between abnormal dendritogenesis and human pathologies and determine if P2X7R plays a regulatory role in the complex pathomechanisms of neurodevelopmental diseases.

## Materials and Methods

### Mouse studies

The studies were conducted in accordance with the principles and procedures outlined in the National Institutes of Health's *Guide for the care and use of laboratory animals* and were approved by the local Animal Care Committee of the Institute of Experimental Medicine (reference #PEI/001/778-6/2015, PE/EA/297-1/2021). Embryonic day (E)17.5-E18.5 *P2rx7*^+/+^ (WT) and *P2rx7*^−/−^ (KO) mouse embryos were used. Homozygous *P2rx7*^+/+^ mice were bred on the C57Bl/6J background. The original breeding pairs of *P2rx7*^−/−^ mice were kindly supplied by Christopher Gabel from Pfizer. The *P2rx7*^−/−^ mice harboured the DNA constructs P2X7-F1 (5′-CGGCGTGCGTTTTGACATCCT-3′) and P2X7-R2 (5′-AGGGCCCTGCGGTTCTC-3′), which were previously shown to inactivate P2X7R (Koványi et al., 2016). All mice were backcrossed onto the C57BL/6N background at least 8-10 times, and experiments were performed with littermates as controls. The animals were housed on a 12 h light-dark cycle under specific pathogen-free conditions and had access to food and water *ad libitum*.

### *In vitro* experimental design

To investigate the impact of P2X7R deficiency on neuronal growth, we bred *P2rx7*^+/+^ (WT) mice and *P2rx7*^−/−^ mice (KO), respectively. To study P2X7R expression and the function of P2X7R under physiological conditions during development, control primary hippocampal neurons were maintained until day *in vitro* (DIV)10, when the dendrites were fully developed, and then transfected. Then, the morphology of these neurons was compared with that of primary neurons with either genetic deficiency or pharmacological blockade of P2X7R by Sholl analysis. The same protocol was used to study WT or KO embryos subjected to MIA to explore the role of P2X7R under pathologic conditions.

### Methods details

#### Isolation of primary neurons from E17.5-E18.5 control and P2X7R KO mouse embryos

Primary hippocampal cells were obtained as previously described (Mut-Arbona and Sperlagh, 2022). Cells were counted in a Neubauer chamber and plated at a density of 150,000 cells/well. The day of plating was considered DIV1. When the confluence of the glial cells reached 90%, cytosine-arabinofuranoside (CAR, 10 μm; Sigma, #C6645) was added to the cultures to stop further proliferation and maintain a neuron-enriched culture system with <10% glial cells (Gao, 2011). On DIV6, one-third of the culture medium was replaced with MEM containing the abovementioned supplements. The cells were kept in a cell culture incubator (37°C in 5% CO_2_, 95% air atmosphere) until they were used for experiments.

### Expression of P2X7R in primary cultures

#### qRT-PCR

To measure the expression of P2X7R in the primary cultures, total RNA isolation was performed using an RNase Plus Mini Kit (QIAGEN 74134, lot #169034695) according to the manufacturer's protocol. Briefly, cells were harvested and homogenized with lysis buffer, and the lysate was transferred to a gDNA eliminator spin column. Then, the cells were mixed with 70% ethanol for further centrifugation (15 s at 10,000 rpm). The mixture was transferred to an RNeasy spin column and centrifuged (15 s at 10,000 rpm). Finally, 30 µl of RNase-free water was added directly to the column, the column was centrifuged to elute the RNA, which was stored at −20°C. RNA concentrations were measured using a Nanodrop 2000c spectrophotometer (Fisher Scientific). The RNA integrity was verified by electrophoretic separation on a 1% agarose gel. Reverse transcription of 1 μg RNA into cDNA was performed using the High-Capacity cDNA Archive Kit (Applied Biosystem) according to the manufacturer's protocol. Previously published primer sequences (Sánchez-Nogueiro et al., 2005) were used to measure the levels of the extracellular and intracellular regions of P2X7R by qRT-PCR. The primer sequences used to amplify the extracellular part were 5′-GCACGAATTATGGCACCGTC-3′ (forward primer) and 5′-ACACCTGCCAGTCTGGATTCCT-3′ (reverse primer) and those used to amplify the intracellular part were 5′-AGGATCCGGAAGGAGTT-3′ (forward primer) and 5′-TAGGGATACTTGAAGCCACT-3′ (reverse primer). The housekeeping gene GAPDH was used for normalization to account for intrawell variability [5′-TTCACCACCATGGAGAGGGC-3′ (forward primer) and 5′-GGCATGGACTGTGGTCATGA-3′ (reverse primer)]. qRT-PCR was performed using the SensiFast SYBR Green No-Rox Kit (Bioline Reagents) according to the manufacturer's protocol in a 10 μl total volume. The reaction mixtures were incubated at 95°C for 20 s and then subjected to 40 cycles of PCR (95°C for 1 s and 60°C for 20 s) in a Quant Studio Real-Time PCR System (Applied Biosystems). The P2X7R fragments amplified by qRT-PCR were separated by electrophoresis on a 1% agarose gel and visualized (data not shown). mRNA expression levels were calculated by the 2^-ΔΔCT^ method. The average cycle threshold (Ct) was obtained for five replicates of each sample from three independent cultures. The mean ΔCt values were normalized to that of GAPDH as an endogenous control. Fold changes in the expression of each sequence were compared with baseline levels (DIV1) after normalization to GAPDH expression. The data were analyzed with Applied Biosystems Relative Quantification (RC) powered by Fisher Scientific Cloud.

#### Western blotting

WT and P2X7R KO cells were lysed in RIPA buffer containing 150 mm NaCl, 50 mm Tris-HCl, pH 7.4, 5 mm EDTA, 0.1% (w/v) SDS, 0.5% sodium deoxycholate, and 1% Triton X-100 containing protease inhibitors (10 mg/ml leupeptin, pepstatin A, 4-(2-aminoethyl) benzenesulfonyl-fluoride and aprotinin) on the indicated days (DIV1-DIV7). Total cell lysates were separated by SDS-PAGE. The protein was then transferred onto nitrocellulose membranes, nonspecific binding was blocked by incubation in 5% nonfat dry milk for 1 h at room temperature, and the membranes were incubated overnight with anti-P2X7R (Alomone Labs, RRID:AB_2040068) and anti-β-actin (Cell Signaling Technology, RRID:AB_330288) primary antibodies at 1:1000. The membranes were incubated with HRP-conjugated secondary antibodies (Cell Signaling Technology) for 1 h at room temperature and were developed using an ECL detection system (Fisher Scientific, Invitrogen). The protein band densities were analyzed by ImageJ software (National Institutes of Health). The P2X7R protein band density was normalized to the density of the band representing total protein (the β-actin band).

#### *In vitro* analysis of P2X7R function with calcium imaging

Cells were plated at a density of 300,000 cells/well. To study neuronal activity, neurons were incubated with medium containing pluronic acid (F-127 P3000MP, 1 μm, previously warmed to 36°C) and Oregon Green 488 BAPTA-1 (OGB-488, Fisher Scientific, O6807, 5 μm) on DIV4. Thirty minutes later, the chamber was loaded with crystals coated with the cells, and the dye-containing medium was replaced with fresh medium previously warmed to 36°C. Twenty minutes later, the spontaneous activity of the cells was recorded for 10 min with the N-STORM Super-Resolution System. The following parameters were used: channel intensity of 38% intensity, 30 ms/Hz, 12-bit (no binning), no delay between frames, 20× magnification. Later, the frames were analyzed with a NIS-Element AR microscope, and the fluorescence intensity change (ΔF/F) was calculated for 2 min after acute application of the agonist 2′(3′)-O-(4-benzoyl benzoyl) adenosine 5′-triphosphate triethylammonium salt (BzATP, Sigma, 1 mm). As a control, a calcium ionophore (Sigma, C7522-5MG, 1 mm) was acutely applied, and the fluorescence intensity change (ΔF/F) was calculated for each individual cell.

#### Quantification of nucleotide and nucleosides contents

The concentration of adenine nucleotides (ATP, ADP, AMP) and adenosine (Ado) released into the culture medium were determined using HPLC. The medium (400 µl) was transferred into a cold Eppendorf tube containing 50 µl of 0.1 m perchloric acid and 10 μm theophylline (as an internal standard). The medium was centrifuged (at 3510 × *g* for 10 min at 0°C-4°C), and the supernatant was kept at −20°C until analysis. Online solid phase extraction coupled to the column-switching technique was applied for quantification of nucleotide content in the samples. HPLC separation was performed by a Shimadzu LC-20 AD Analytical System using UV (Agilent 1100 VW, set at 253 nm) detection. A phenyl-hexyl packed (7.5 × 2.1 mm) column was used for online sample enrichment, and separation was completed by coupling to an analytical C-18 (150 × 2.1 mm) column. The flow rates of the mobile phases [Phase A: 10 mm potassium phosphate buffer with 0.25 mm EDTA; Phase B: additional components, such as 0.45 mm octane sulfonyl acid sodium salt, 8% acetonitrile (v/v), 2% methanol (v/v), pH 5.55] were 350 and 450 µl/min, and they were applied in a step gradient (Baranyi et al., 2006). The sample enrichment flow rate of buffer A was 300 µl/min for 4 min, and the total run time was 55 min. Concentrations were calculated by an internal standard 2 point calibration curve method [*(A_i_ × f × B)/(C × D_i_*), where *A_i_* is the area of nucleotide components, *B* is the sample volume, *C* is the injection volume, *D_i_* is the response factor for 1 pmol nucleotide standard, and *f* is the international standard factor (IS area in the calibration/IS area in the actual sample)]. The data are expressed as pmol per ml.

### Branching analysis for primary hippocampal cells

#### eGFP transfection

The plasmid pAVV-Syn-GFP (Addgene 58867, RRID:Addgene_58867), in which GFP is expressed under the control of the Synapsin promoter, was transfected into cells at DIV10 to allow clear visualization of the morphology of individual neurons. Neurolucida Software was used to study the differences in dendritic outgrowth in neurons from mice of the different genotypes in a blinded manner as described previously (Mut-Arbona and Sperlagh, 2022). The number of intersections was determined every 2 μm starting at a radius of 5 μm from the center of the neuronal soma.

#### Immunocytochemistry (ICC)

After fixation, the cells were permeabilized in PBS/0.1% Triton X-100 (for 12 min). Then, they were blocked in 3% BSA/PBS for 30 min. For MAP2 staining, an α-MAP2 (Sigma, M9942, RRID:AB_477256) primary antibody was used at a 1:1000 dilution, and an AlexFluor-594-conjugated anti-mouse secondary antibody (Invitrogen, RRID:AB_2762825) was used at a 1:500 dilution. For GFAP staining, an α-GFAP antibody (SYSY, RRID:AB_10641162) was used at a 1:1000 dilution, and an AlexaFluor-647-conjugated anti-guinea pig secondary antibody (Invitrogen, RRID:AB_2735091) was used at a 1:500 dilution. For P2X7R staining, cells were incubated with a rabbit α-P2X7 antibody (Alomone Labs, RRID:AB_2040068) diluted 1:100 for two nights and then with AlexaFluor-549-conjugated anti-rabbit (Invitrogen, RRID:AB_2762824) and AlexaFluor-488-conjugated anti-rabbit (Invitrogen, RRID:AB_2633280) secondary antibodies diluted 1:500. To enhance the GFP signal for morphologic analysis of primary hippocampal neurons, immunostaining was performed. Cells were incubated with an α-GFP primary antibody (Aves Labs, RRID:AB_10000240) diluted 1:1000 overnight at 4°C followed by an AlexaFluor-488-conjugated anti-chicken secondary antibody (Invitrogen, RRID:AB_2534096) diluted 1:500. Nuclei were counterstained with 1 nm Hoechst solution (Invitrogen, #5117) before mounting. Images were taken with a Nikon C2^+^ confocal microscope connected to a Nikon Ni-E fully automatized microscope using a 20× objective. Image acquisition was performed using NIS Elements 4.3 software (RRID:SCR_014329).

### Hippocampal slice experiment

#### Slice preparation and biocytin labeling

Hippocampal slices were prepared from 20- to 29-d-old WT and *P2rx7*^−/−^ mice on the C57Bl/6J background. The mice were anesthetized with Forane (4 g/μl) and decapitated. The brains were quickly removed and cut into 300 μm transverse slices using a vibratome (Leica VT1200) in ice-cold cutting solution (in mm as follows: 85 NaCl, 2.5 KCl, 0.5 CaCl, 1.25 NaH_2_PO_4_, 24 NaHCO_3_, 25 glucose, and 75 sucrose, pH 7.4, 290-300 mOsm) bubbled with 95% O_2_ and 5% CO_2_. After 30 min of incubation at 34°C, the slices were stored at room temperature in ACSF (in mm as follows: 126 NaCl, 2.5 KCl, 2 CaCl_2_, 2 MgCl_2_, 1.25 NaH_2_PO_4_, 26 NaHCO_3_, and 10 glucose, pH 7.4, 300-310 mOsm) bubbled with 95% O_2_ and 5% CO_2_. Pyramidal cells were visualized using differential interference contrast microscopy with an Olympus BX50WI microscope and a 40× water immersion objective (LUMPLFLN 40×WI, 0.8 NA; Olympus). Cells were patched using borosilicate glass pipettes with a resistance of 4-7 mΩ. After 15 min, the recording pipette was quickly retracted from the cell to help maintain cell integrity for histology. The slices were then fixed overnight with 4% PFA at 4°C.

#### AMPA/NMDA ratio

Bipolar electrodes (World Precision Instruments) were used to stimulate Schaffer collaterals and patch pyramidal cells in the CA1 region. The internal solution contained the following (in mm): 115 Cs-methanesulfonate, 8 NaCl, 10 HEPES, 0.3 Na-GTP, 4 Mg-ATP, 10 PO creatine, 8 biocytin, 5 QX314, adjusted to a pH of 7.2 with CsOH. Gabazine (10 μm) was added to the ACSF to block GABA_A_ receptors. A holding potential of −70 mV was used to record AMPA components, as GABA_A_ receptors were pharmacologically blocked and NMDA components cannot be recorded at −70 mV in normal ACSF because of the voltage-dependent blockade of Mg^2+^. The neurons were then held at 40 mV to record NMDA-mediated EPSCs. Because AMPA components could also be evoked at 40 mV, we only considered the NMDA component amplitude 50 ms after the peak at 40 mV, when the AMPAR component returned to baseline (Kasanetz and Manzoni, 2009; Miwa et al., 2011). The AMPA/NMDA ratio was determined by calculating the AMPA current amplitude relative to the NMDA current amplitude at 50 ms after stimulus (average of 10-15 sweeps).

#### Immunostaining of biocytin-filled cells

The day after electrophysiological recordings, the slices were washed with 0.1 m PBS, followed by 0.05% TBS, and then blocked with 5% NGS (Vector Laboratories) containing 0.03% Triton X-100 for 30 min. The slices were subsequently incubated in AlexaFluor-564-conjugated streptavidin (S11227, Invitrogen), diluted 1:500 in 0.03% Triton X-100 (Invitrogen) for at least 4 h. After washing with TBS and PBS repeatedly, the slices were mounted on gelatin-coated glass slides using Fluoroshield mounting medium (F6182-20ML, Sigma). The examined cells were morphologically analyzed based on images taken with a Nikon C2^+^ confocal microscope connected to a Nikon Ni-E fully automatized microscope using a 20× objective. Image acquisition was performed using NIS Elements 4.3 software.

#### Spine density

To visualize dendritic spines, 300-μm-thick biocytin-labeled slices were removed from the slides and stored in 0.1 mm PB. After three washes with 0.1 mm PB, the slices were embedded in 2% agarose gels and further cut into 50 μm sections with a vibratome (Leica VT1200s). Then, the 50 μm slices were remounted on slides with Fluoroshield and visualized with a Nikon C2 confocal microscope and 60× oil immersion objective (NA 1.4) at a pixel size of 0.07. *Z* stacks were acquired at a step interval of 0.125 μm. Images of the molecular layer of the inner dentate gyrus were taken. The original images were further deconvoluted by using Huygens Professional version 19.04 (Scientific Volume Imaging; http://svi.nl) before quantification. Then, the deconvoluted images were imported into Neurolucida software (MicroBrightField, RRID:SCR_001775) to trace the spines and quantify the total number of spines per 30 µm.

#### Cognitive performance of young animals

The cognition of young animals were tested according to the method described by Cruz-Sanchez et al. (2020) with minor modifications. Briefly, all recognition tests were performed in a white squared Plexiglas chamber (40 × 40 × 40). The mice were handled and habituated to the chamber from postnatal day (P)20 to the testing day of each of the three recognition memory tasks. During the handling and habituation phase, the animals were handled for 5 min and then habituated to the empty chamber for 5 min. Males and females underwent different cognitive tests on P21, P24, P25, and P28. The mice were assigned randomly to experimental cohorts to prevent impairment of cognitive performance because of repeated testing. The first cohort underwent the object location (OL) task (P21) and temporal object recognition (TOR) task (P28), while the second cohort underwent only the novel object recognition (NOR) task (P24 and P25).

### Behavioral tests

The tests were performed with The Observer XT 12 (Noldus) by an experimenter blinded to the experimental conditions. The discrimination index was calculated for all the tests as a measure of novelty preference by dividing the time spent exploring the novel location/novel object/familiar object by the sum of the time spent exploring both objects.

For all tests in Cohort 1, during the exploration phase, the animals were allowed to explore two identical objects for 10 min and then subjected to a 1 h interphase interval. In the OL task, the mice underwent a 5 min test phase in which one of the objects was relocated to a novel location opposite the original one (see Fig. 4*M*). During the 5 min test phase of the TOR test, the mice were allowed to explore one familiar object (the object from exploration Phase 1) and one novel object (the object from exploration Phase 2) (see Fig. 4*Q*).

The animals in Cohort 2 were allowed to explore two identical objects until they explored the objects for a total of 20 s. Then, the animals were placed back in their home cages. Immediately (after <2 min), the mice underwent a 5 min test phase in which the familiar object and a novel object were presented. The animals were subjected to another 5 min test phase in the same chamber again after a longer delay (24 h), in which a familiar object and a novel object different from the previously used object were presented (see Fig. 4*N*).

### MIA model: experimental design

An MIA protocol was used to specifically trigger schizophrenia-like behavior in offspring (Vigli et al., 2020). Intraperitoneal poly(I:C) (PIC) injections during pregnancy activate antiviral pattern recognition receptors, such as TLR3 and PKR, in the mother and thereby activate the maternal immune system. For the *in vitro* experiments, pregnant WT and KO mice were randomly assigned to different treatment groups and injected intraperitoneally with 10 mg/kg or 20 mg/kg PIC on E12.5. Control mice received saline injection (100 µl) at the same time point. Primary hippocampal cells were obtained from embryos of both genotypes from control and PIC-treated animals on E17.5-E18.5 and processed as described previously for morphologic studies. Second, pregnant WT and KO mice were randomly assigned to different treatment groups and intraperitoneally injected with a higher dose of PIC, 20 mg/kg on E12.5 for behavioral studies. Control mice received saline injection (100 µl) at the same time point. After they reached 4 weeks of age, the offspring of control and immune-activated animals were weaned and house in cages containing 2-4 animals. Test-naive male mice were subjected to behavioral experiments in the same order (open field, T maze, NOR, social preference, and prepulse inhibition [PPI] test) at 8-10 weeks of age by an experimenter blinded to the treatment groups.

### Quantitative analysis of IL-1β using ELISA

To measure the possible inflammatory changes in the MIA model, IL-1β levels for E13.5 fetal brains were measured by the Quantikine Mouse IL-1β Immunoassay (R&D Systems). Fetal brains were collected 24 h after intraperitoneal injection of PIC (20 mg/kg) or saline treatment from pregnant mice. For homogenization, samples were placed in lysis buffer (50 mm Tris HCl, 150 mm NaCl, 5 mm CaCl_2_, 0.02% NaN_2_, 1% Triton X-100, pH 7.4) with 0.1% protease inhibitor double-diluted in PBS. After homogenization and centrifugation, brain homogenates were stored at −80°C. IL-1β concentration was determined by mouse IL-1β-specific monoclonal antibody precoated microplates following the manufacturer's instructions. We determined the optical density with a microplate reader at 450 nm (Cytation5 Cell Imaging Multi-Mode Reader). We calculated concentration values (pg/ml) using GraphPad. For measuring total protein level in tissue samples, absorbance was measured at 560 nm. Fetal brain IL-1β concentrations were expressed in pg/mg protein.

### Behavioral tests

#### Open field test

Mice were placed in the middle of a white square box (40 × 40 cm), and the Ethovision XT 15 system (Noldus, RRID:SCR_000441) connected to an overhead camera was used to record and measure locomotor activity for 10 min. The total distance traveled during the analysis was measured in centimeters. Velocity and percentage of movement were also measured during the test.

#### T-maze and NOR tests

Spatial working memory and NOR were tested according to the method described by Castañé et al. (2015) with minor modifications. Briefly, mice were habituated to a T-maze (arm: 30 × 20 cm) for 10 min. The percentage of successful alternations was evaluated in the first 3 min. Based on their innate curiosity, mice tend to enter all three arms of the T-maze successively, and these successive entries constitute a spontaneous alternation. An arm entry occurred when all four paws of a mouse crossed the threshold of the central zone and entered the corresponding arm. The spontaneous alternation percentage was calculated as follows:

$$Spontaneousalternation\%=\frac{Numberofspontaneousalternations}{Totalnumerofentries-1}\times 100$$

Twenty-four hours later, NOR test was performed. The total time spent exploring each of the two objects (the novel and familiar object) in 5 min was recorded by the Ethovision XT 15 system (Noldus). The location of the novel object was alternated across trials. The NOR index (%) was calculated as follows (the time the test mouse spent interacting with the novel object [*t_n_*] divided by the total time the test mouse spent interacting with the novel and the familiar object [*t_f_*] multiplied by 100) as follows:

$$Novelobjectrecognition\%=\frac{t_{n}}{t_{n}+t_{f}}\times 100$$

#### Social preference test

Social preference was assessed according to a previously described method (Horváth et al., 2019). An Ethovision XT 15 system (Noldus) connected to an overhead camera was used to track and record the time each test mouse spent in each of the sniffing zones. The location of the stranger mouse was alternated across trials. Social preference is presented as the cumulative time (seconds) spent in every chamber.

#### Acoustic startle

Baseline startle magnitude, PPI, and habituation to startle were tested using startle chambers (San Diego Instruments). Five blocks were included in the session. First, a 5 min acclimatization period was followed by the presentation of five 120 dB startle pulses (Block 1). In Block 2, the startle response was tested by presenting randomly generated pulses of different intensities (80, 90, 100, 110, and 120 dB). For measurement of PPI, three prepulse intensities (68, 71, and 77 dB) were preceded by 120 dB pulses (Block 3). In Block 4, the interstimulus interval was tested by presenting 73 dB prepulses 25, 50, 100, 200, or 500 ms before 120 dB pulses. Finally, in Block 5, five 120 dB pulses were delivered to assess startle habituation. In all experiments, the average startle magnitude over the recording window (65 ms) was used for all data analyses. Startle pulses were 40 ms in duration, prepulses were 20 ms in duration, and prepulses preceded pulses by 100 ms (onset–onset). A 65 dB background was presented continuously throughout the session. The house light remained on throughout all testing sessions.

### Drugs and treatments

The following drugs were used in our experiments: polyinosinic–polycytidylic potassium salt (Sigma, P9582-5MG, batch #0000080809), JNJ47965567 (Tocris Bioscience), ITH15004 (personal contribution from Instituto Teófilo Hernando), and ARL 67156 trisodium salt (Tocris Bioscience, 1283, batch #16A/253182). All drugs were dissolved in sterile saline, except for JNJ47965567 and ITH15004, which were dissolved in 30% Captisol solution (sulfobutyl ether-7-cyclodextrin) and 5% DMSO, respectively.

### Statistical analyses

Based on previous experience, one pregnant dam gives birth to an average of 10 infants; therefore, we calculated the ideal sample size before the breeding process as described previously (Horváth et al., 2019). Statistical analyses were performed using STATISTICA version 64 (StatSoft, RRID:SCR_014213) and Graph Prism version 8.0.2 (GraphPad Software, RRID:SCR_002798). Data normality was tested using the Kolmogorov–Smirnov test and Shapiro–Wilk test. Possible outlier values were detected by the ROUT method (*q* = 1%) (Motulsky and Brown, 2006) and omitted from the analysis. Differences were analyzed using Student's unpaired *t* test for two groups, one-way ANOVA followed by the Kruskal–Wallis *post hoc* or Dunn's multiple comparisons test, or two-way ANOVA followed by Bonferroni's *post hoc* test or the Mann–Whitney *U* test for multiple comparisons, as appropriate. The statistics of all the molecular and morphologic experiments are specified in Table 1, while the results obtained in behavioral experiment are summarized in Table 2. All data are expressed as the mean ± SEM. *p* < 0.05 was considered statistically significant.

**Table 1.** Statistical analysis for molecular and morphologic analysis

Experiment and figure		Statistical analysis	Interaction value	*p*	*Post hoc*	*p*	*n*
qPCR	1*C*,*D*	Ordinary one-way ANOVA	*F*_(9, 39)_ = 2.616	0.0181	Sidak's multiple comparisons test		3, 5, 3, 5, 6, 6, 4, 5, 5, 7
					DIV1 vs Div4	0.0449	3, 5
		Ordinary one-way ANOVA	*F*_(9, 39)_ = 3.284	0.0046	Sidak's multiple comparisons test		5, 5, 3, 5, 6, 6, 4, 5, 5, 5
					DIV1 vs Div4	0.0088	5, 5
					DIV1 vs Div7	0.0458	5, 4
Western blot	1*E*,*F*	Ordinary one-way ANOVA	*F*_(6, 21)_ = 2.367	0.0665	Dunnett's multiple comparisons test		4, 4
					DIV1 vs DIV4	0.0407	
		Ordinary one-way ANOVA	*F*_(5, 18)_ = 6.048	0.0019	Tukey's multiple comparisons test		4, 4, 4, 4, 4, 4
					DIV1WT vs DIV4WT	0.0065	
					DIV4WT vs DIV7WT	0.0305	
					DIV4WT vs DIV4KO	0.0039	
Calcium imaging	1*G*,*H*	Mann–Whitney test		<0.0001			150, 196
		Mann–Whitney test					135, 196
Sholl analysis	2 *D*	Two-way ANOVA	*F*_(191, 21696)_ = 25.33	<0.0001	Bonferroni's multiple comparisons test		55, 60
					Radius 15 μm to 193 μm	<0.0001	
	2F	Mann–Whitney test		<0.0001			55, 60
	2 *G*	Unpaired *t* test		<0.0001			55, 60
	2 *H*	Unpaired *t* test		<0.0001			55, 60
	2 *I*	Unpaired *t* test		<0.0001			55, 60
HPLC	2 *E*	Two-way ANOVA	*F*_(1, 36)_ = 125.4	<0.0001	Sidak's multiple comparisons test		4, 7
					ATP	<0.0001	
					ADP	<0.0001	
					AMP	0.0558	
					Ado	<0.0001	
Sholl analysis with P2X7R antagonists	3 *B*	Two-way ANOVA	*F*_(292, 15835)_ = 4.231	<0.0001	Bonferroni's multiple comparisons test		45, 33, 35
					WT vs ITH15005 Radius 17 μm to 119 μm	<0.0001	
					WT vs JNJ Radius 21 μm to 143 μm	<0.0001	
	3 *C*	Ordinary one-way ANOVA	*F*_(2, 105)_ = 0.5159	0.5984			45, 33, 35
	3 *D*	Ordinary one-way ANOVA	*F*_(2, 105)_ = 8.158	0.0005	Sidak's multiple comparisons test		45, 33, 35
					WT vs ITH15005	0.0085	
					WT vs JNJ	0.0006	
	3 *E*	Ordinary one-way ANOVA	*F*_(2, 105)_ = 19.31	<0.0001	Sidak's multiple comparisons test		45, 33, 35
					WT vs ITH15005	<0.0001	
					WT vs JNJ	<0.0001	
	3 *F*	Ordinary one-way ANOVA	*F*_(2, 105)_ = 4.194	0.0177	Sidak's multiple comparisons test		45, 33, 35
					WT vs ITH15005	0.0096	
Sholl analysis with ARL	3 *H*	Two-way ANOVA	*F*_(157, 9954)_ = 1252	0.0184	Bonferroni's multiple comparisons test		30, 35
					Radius 151 μm to 159 μm	0.0025	
	3 *J*	Unpaired *t* test		0.8527			30, 35
	3 *K*	Unpaired *t* test		0.2820			30, 35
	3 *L*	Unpaired *t* test		0.0140			30, 35
	3 *M*	Unpaired *t* test		0.0009			30, 35
HPLC ARL	3 *I*	Two-way ANOVA	*F*_(2, 36)_ = 37,64	<0.0001	Sidak's multiple comparisons test		7,7
					ATP	0,000548	
					ADP	0,036417	
					AMP	1,000000	
Sholl analysis pyramidal neurons	4 *B*	Two-way ANOVA	*F*_(407, 12648)_ = 2.156	<0.0001	Bonferroni's multiple comparisons test		16, 17
					Radius 17 μm to 85 μm	<0.0001	
	4 *C*	Mann–Whitney test		0.6048			16, 17
	4 *D*	Mann–Whitney test		0.2768			16, 17
	4 *E*	Mann–Whitney test		0.002			16, 17
	4 *F*	Mann–Whitney test		0.0326			16, 17
	4 *H*	Two-way ANOVA	*F*_(250, 5020)_ = 0.2482	>0.9999			12, 10
	4 *I*	Mann–Whitney test		0.2614			12, 10
	4 *J*	Mann–Whitney test		0.3066			12, 10
	4 *K*	Mann–Whitney test		0.8085			12, 10
	4 *L*	Mann–Whitney test		0.7354			12, 10
Synaptic strength	5 *B*	Unpaired *t* test		0.5759			11, 10
	5 *C*	Unpaired *t* test		0.0065			11, 10
	5 *D*	Unpaired *t* test		0.0015			11, 10
	5 *E*	Unpaired *t* test		0.872			11, 10
	5 *F*	Unpaired *t* test		0.3835			11, 10
	5 *G*	Unpaired *t* test		0.7068			11, 10
	5 *H*	Unpaired *t* test		0.3835			11, 10
	5 *I*	Unpaired *t* test		0.0003			22, 18
Sholl analysis PIC	6 *A*	Two-way ANOVA	*F*_(654, 40058)_ = 13.92	<0.0001	Bonferroni's multiple comparisons test		55, 46, 39, 50
					WT vs 10 from radius 13 μm to 201	<0.0001	
					WT vs 20 from radius 13 μm to 223 μm	<0.0001	
	6 *B*	Two-way ANOVA	*F*_(444, 22052)_ = 0.5251	>0.9999			55, 46, 39, 50
	6 *C*	Ordinary one-way ANOVA	*F*_(3, 186)_ = 6.703	0.0003	Sidak's multiple comparisons test		55, 46, 39, 50
					WT vs 20	<0.0001	
	6 *D*	Ordinary one-way ANOVA	*F*_(3, 185)_ = 25.29	<0.0001	Sidak's multiple comparisons test		55, 46, 39, 50
					WT vs SAL	<0.0001	
					WT vs 10	<0.0001	
					WT vs 20	<0.0001	
	6 *E*	Ordinary one-way ANOVA	*F*_(3, 186)_ = 55.76	<0.0001	Sidak's multiple comparisons test		55, 46, 39, 50
					WT vs SAL	0.0002	
					WT vs 10	<0.0001	
					WT vs 20	<0.0001	
	6 *F*	Ordinary one-way ANOVA	*F*_(3, 186)_ = 29.50	<0.0001	Sidak's multiple comparisons test		55, 46, 39, 50
					WT vs 10	<0.0001	
					WT vs 20	<0.0001	
	6 *G*	Ordinary one-way ANOVA	*F*_(3, 148)_ = 1.568	0.1997			
	6 *H*	Ordinary one-way ANOVA	*F*_(3, 148)_ = 0.3550	0.7856			
	6 *I*	Ordinary one-way ANOVA	*F*_(3, 148)_ = 9.464	0.0001	Sidak's multiple comparisons test		
					KO vs 10	0.0005	
					KO vs 20	0.0006	
	6 *J*	Ordinary one-way ANOVA	*F*_(3, 148)_ = 2.802	0.0420			
HPLC	6 *K*	Two-way ANOVA	*F*_(9, 112)_ = 72.10	<0.0001	Sidak's multiple comparisons test		7, 12, 7, 6
					ATP WT vs 20	<0.0001	
					ATP WT vs 10	<0.0001	
					ADP WT vs 10	0.0166	
	6 *L*	Two-way ANOVA	*F*_(9, 96)_ = 18.84	<0.0001	Sidak's multiple comparisons test		4, 4, 12, 8
					ATP KO vs 20	<0.0001	
					ATP KO vs 10	<0.0001	
					ADP KO vs SAL	0.0056	
					AMP KO vs 10	0.0062	
					ADO KO vs 20	0.0008	
					ADO KO vs 10	0.0004	
ELISA	7 *B*	Two-way ANOVA			Sidak's multiple comparisons test		
		Interaction	*F*_(1, 43)_ = 1897	0.1755	WT SAL vs WT PIC	0,0050	7, 13, 16,11
		Treatment	*F*_(1, 43)_ = 1393	0.2444			
		Genotype	*F*_(1, 43)_ = 12,58	0.0010			

**Table 2.** Behavioral statistical analysis

Experiment and figure		Statistical analysis	Interaction value	*p*	*Post hoc*	*p*	*n*
OL recognition	4 *O*	No sex differences: two-way ANOVA [*F*_(1, 23)_ = 0.6508, *p* = 0.4281]	14, 13				
		Unpaired *t* test		<0.0001			
NOR	4 *R,S*	No sex differences: two-way ANOVA [*F*_(1, 21)_ = 0.2054, *p* = 0.655]	13, 13				
		Mann–Whitney test		<0.0001			
		No sex differences: two-way ANOVA [*F*_(1, 22)_ = 0.2653, *p* = 0.6117]	13, 13				
		Mann–Whitney test		<0.0001			
TOR	4 *P*	No sex differences: two-way ANOVA [*F*_(1, 23)_ = 0.5439, *p* = 0.4683]	14, 13				
		Mann–Whitney test		0.0047			
Open field	7 *C*	No sex differences: two-way ANOVA [*F*_(1, 23)_ = 0.5439, *p* = 0.4683]	19, 22, 20, 17				
		Two-way ANOVA					
		Interaction	*F*_(1, 74)_ = 0.02201	0.8825			
		Treatment	*F*_(1, 74)_ = 5.489	0.0218			
		Genotype	*F*_(1, 74)_ = 0.7304	0.3955			
	7 *D*	No sex differences: Three-way ANOVA [*F*_(1, 69)_ = 3.254, *p* = 0.0756]					
		Two-way ANOVA					19, 22, 20, 17
		Interaction	*F*_(1, 74)_ = 0.04155	0.839			
		Treatment	*F*_(1, 74)_ = 6.155	0.0154			
		Genotype	*F*_(1, 74)_ = 0.7932	0.376			
T-maze and NOR	7 *E*	No sex differences: Three-way ANOVA [*F*_(1, 69)_ = 2.074 *p* = 0.1544]	19, 22, 20, 17				
		Two-way ANOVA			Sidak's multiple comparisons test		
		Interaction	*F*_(1, 73)_ = 2.295	0.1341	WT vs 20	0.0224	
		Treatment	*F*_(1, 73)_ = 4.477	0.0378			
		Genotype	*F*_(1, 73)_ = 5.774	0.0188			
	7 *F*	No sex differences: Three-way ANOVA [*F*_(1, 69)_ = 1.564, *p* = 0.2153]	19, 22, 20, 17				
		Two-way ANOVA			Sidak's multiple comparisons test		
		Interaction	*F*_(1, 74)_ = 4.179	0.0445	WT vs 20	0.0251	
		Treatment	*F*_(1, 74)_ = 2.419	0.1241			
		Genotype	*F*_(1, 74)_ = 0.002605	0.9594			
Three chamber social test	7 *G*	No sex differences: three-way ANOVA [*F*_(1, 69)_ = 0.4612 *p* = 0.4993]	19, 22, 20, 17				
		Two-way ANOVA					
		Interaction	*F*_(1, 74)_ = 0.05707	0.8118			
		Treatment	*F*_(1, 74)_ = 0.003845	0.9507			
		Genotype	*F*_(1, 74)_ = 1.082	0.3016			
Startle acoustic test	7 *H*	No sex differences: three-way ANOVA [*F*_(1, 63)_ = 0.4812, *p* = 0.4904]	18, 22, 20, 17				
		Two-way ANOVA			Sidak's multiple comparisons test		
		Interaction	*F*_(1, 73)_ = 0.3223	0.572	WT vs 20	0.0309	
		Treatment	*F*_(1, 73)_ = 8.677	0.0043			
		Genotype	*F*_(1, 73)_ = 0.7274	0.3965			
		No sex differences: three-way ANOVA [*F*_(1, 63)_ = 0.04105, *p* = 0.8401]	18, 22, 20, 17				
		Two-way ANOVA					
		Interaction	*F*_(1, 73)_ = 0.1835	0.6696			
		Treatment	*F*_(1, 73)_ = 2.264	0.1367			
		Genotype	*F*_(1, 73)_ = 0.5829	0.4476			
		No sex differences: three-way ANOVA [*F*_(1, 63)_ = 1.181, *p* = 0.2814]	18, 22, 20, 17				
		Interaction	*F*_(1, 73)_ = 0.05067	0.8225			
		Treatment	*F*_(1, 73)_ = 2.309	0.133			
		Genotype	*F*_(1, 73)_ = 0.5806	0.4485			
PPI	7 *I*						18, 22, 20, 17
		No sex differences: three-way ANOVA [*F*_(1, 63)_ = 1.777, *p* = 0.1874]					
		Two-way ANOVA			Sidak's multiple comparisons test		
		Interaction	*F*_(1, 73)_ = 2.447	0.122	WT vs 20	0.026	
		Treatment	*F*_(1, 73)_ = 4.173	0.0447			
		Genotype	*F*_(1, 73)_ = 1.694	0.1971			
		No sex differences: three-way ANOVA [*F*_(1, 63)_ = 0.5386, *p* = 0.4657]					
		Two-way ANOVA			Sidak's multiple comparisons test		
		Interaction	*F*_(1, 73)_ = 1.725	0.1932	WT20 vs KO_2_0	0.0009	
		Treatment	*F*_(1, 73)_ = 0.6170	0.4347			
		Genotype	*F*_(1, 73)_ = 15.78	0.0002			
		No sex differences: three-way ANOVA [*F*_(1, 63)_ = 0.002469, *p* = 0.9605]					
		Two-way ANOVA			Sidak's multiple comparisons test		
		Interaction	*F*_(1, 74)_ = 1.449	0.2325	WT vs 20	0.0054	
		Treatment	*F*_(1, 74)_ = 10.04	0.0022	WT20 vs KO_2_0	0.0026	
		Genotype	*F*_(1, 74)_ = 13.30	0.0005			

### Data availability

This study includes no data deposited in external repositories.

## Results

### P2X7R expression is decreased during development *in vitro*

**Figure 1.**
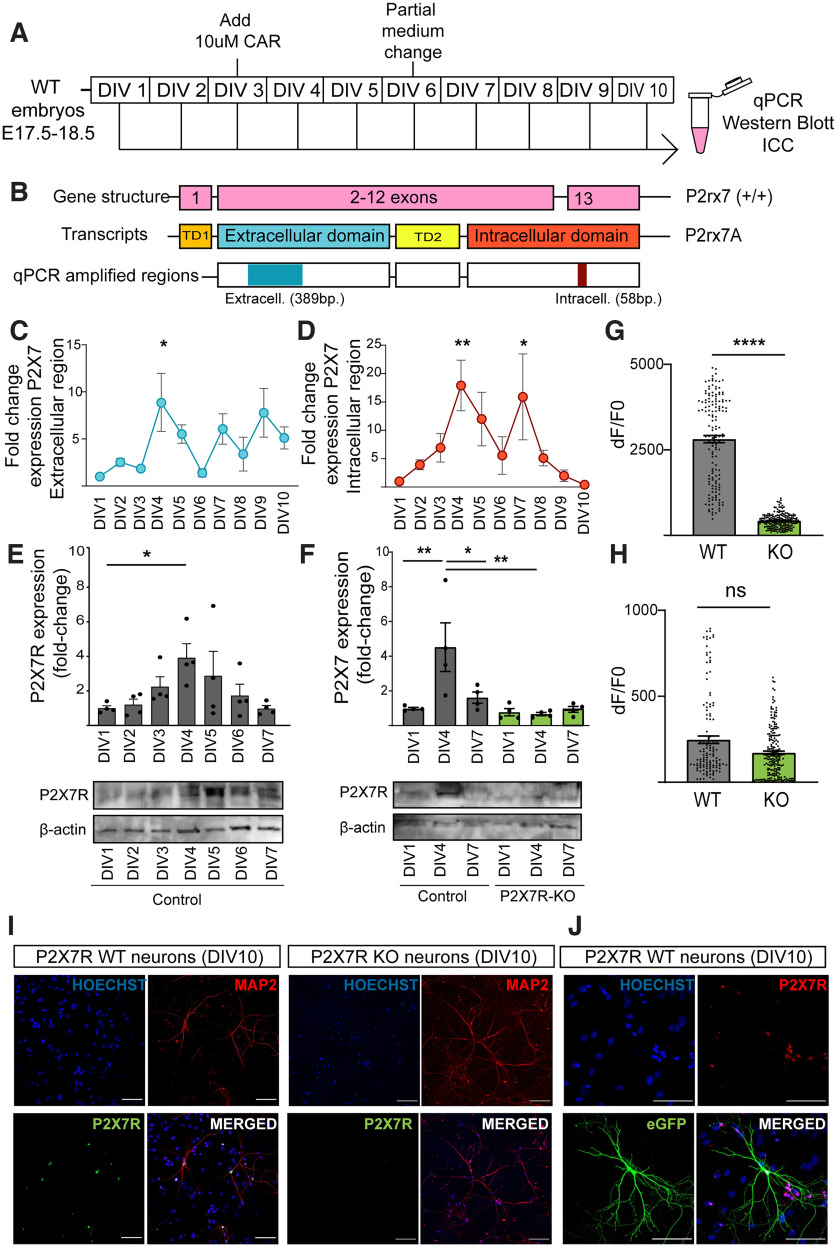
P2X7R is present in neurons cultivated *in vitro* during development. ***A***, Scheme of the experiment. ***B***, Scheme of the regions amplified by qPCR. ***C***, Analysis of the expression of extracellular and (***D***) intracellular sequences of P2X7R by real-time qPCR (DIV1) on each DIV. Relative expression is presented as the fold change normalized to GAPDH expression. ***E***, WT cells were cultured for the indicated period of time, and P2X7R protein expression was determined by immunoblotting. Graphs represent densitometric evaluation of the target protein band normalized to the total amount of loaded protein (*n* = 4). ***F***, To validate the specificity of the P2X7R antibody, WT and P2X7R-KO cells were cultured for the indicated period of time, and P2X7R protein expression was determined by immunoblotting. Graphs represent the densitometric evaluation of the target protein band normalized to total amount of loaded protein (*n* = 4). ***G***, Areas under the curve for WT and KO primary pyramidal cells at DIV4 after acute BzATP (1 mm) application. ***H***, Areas under the curve for WT and KO primary pyramidal cells at DIV4 after acute calcium ionophore (1 mm) application. ***I***, Immunostaining of a neuronal marker (MAP2) and P2X7R in KO and WT primary neurons at DIV10. Nuclei were counterstained with Hoechst. Scale bar, 100 µm. ***J***, Immunostaining for a transfection marker (eGFP) and P2X7R at DIV10. Nuclei were counterstained with Hoechst. Scale bar, 200 µm. *p < 0.05. **p < 0.005. ****p < 0.0001. ns, non-significant. Data presented as Mean±SEM.

To study P2X7R expression, RNA was collected each day for 10 d. To assess the role of P2X7R in neuronal growth, the indirect effect of astroglia on neurons was blocked by adding CAR (10 μm), an anti-mitogen agent, to the cultures on DIV3 (Fig. 1*A*). Then, the transcript level of the *P2rx7* gene was measured by qRT-PCR (Fig. 1*B*), and the results revealed changes in the average expression of both the intracellular and extracellular regions during the first days of culture (Fig. 1*C*,*D*). qRT-PCR revealed that there was a significant increase in the average transcript level of the extracellular region on DIV4 (Fig. 1*C*), and we observed a similar tendency for the intracellular regions. Notably, the level of the intracellular region increased during the first days of culture, corresponding to the growth of the neurons, and transiently increased at DIV7 (i.e., after the medium change) but was reduced immediately after and dropped below the initial level (Fig. 1*D*). Additionally, consistent with the real-time PCR results, the protein expression of P2X7R was transiently increased, with P2X7R expression gradually increasing from DIV3, reaching a peak at DIV4 and subsequently declining to a level below the detection limit at DIV7 (Fig. 1*E*). The specificity of the P2X7R antibody was validated using cells harvested from WT and P2X7R KO animals (Fig. 1*F*). To study the functional activity of the receptor *in vitro*, we performed calcium imaging at DIV4, at which point the expression of the receptor was higher. We observed that neurons from WT mice responded to BzATP (1 mm), an agonist of P2X7R, while the response of primary neurons from KO mice was negligible (Fig. 1*G*). As a control, we used a calcium ionophore (1 mm); neurons from both genotypes presented the same response to this compound (Fig. 1*H*).

To study the effects of P2X7R on branching and neuronal development, we cultivated neurons until DIV10, at which point the dendrites were mostly fully developed. We assessed the presence of P2X7R in our cultures at DIV10 and found that it was expressed on neurons *in vitro* (Fig. 1*I*). To study the morphology of individual neurons, we benefited from the lower effectivity of the transfection on cultured wells and performed ICC for visualization and tracing. Specifically, cells were transfected with a plasmid encoding GFP for this purpose. Again, P2X7R was detected specifically in transfected neurons by immunostaining (Fig. 1*J*).

### P2X7R regulates dendritic outgrowth in neuronal cultures

**Figure 2.**
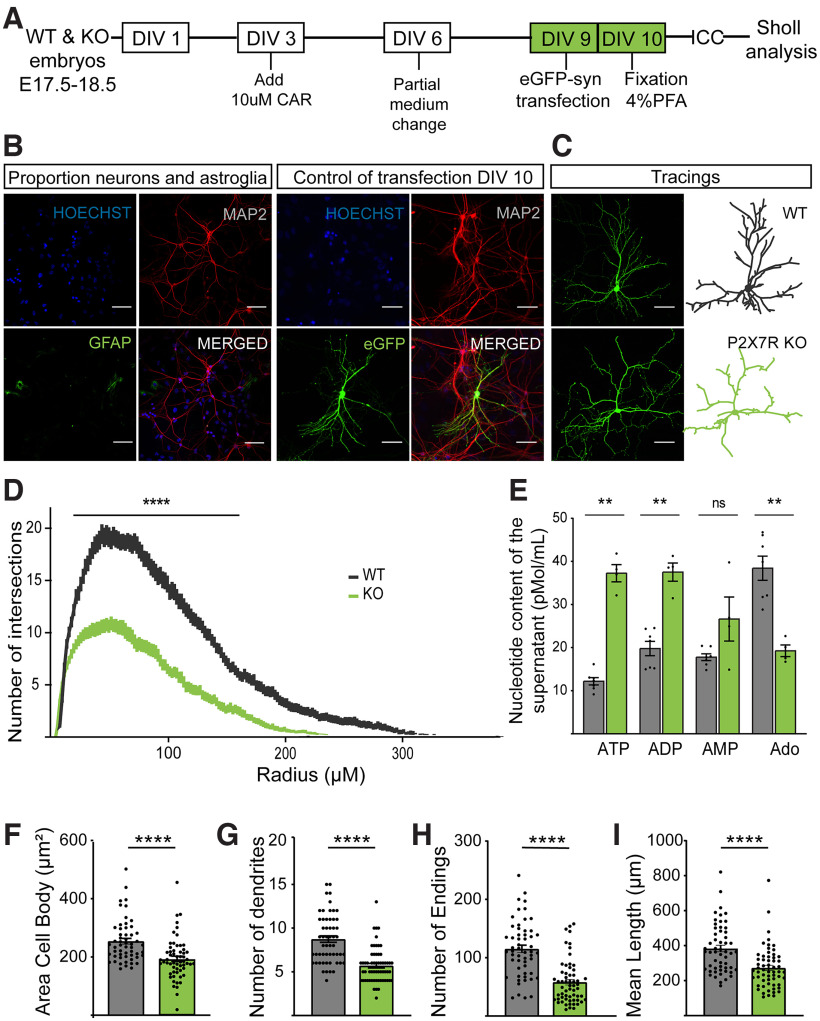
Neurons from *P2rx7*^−/−^ mice present less dendritic outgrowth. ***A***, Scheme of the *in vitro* experiment. ***B***, Immunostaining of a neuronal marker (MAP2) and an astroglial marker (GFAP) and of a transfection marker (eGFP) and a neuronal marker (MAP2) as the control for the transfection in a representative control WT primary hippocampal neuron at DIV10. Nuclei were counterstained with Hoechst. Scale bar, 200 µm. ***C***, Representative hippocampal neurons from WT and P2X7R KO mice transfected with and stained for eGFP. ***D***, Sholl analysis showing the number of intersections versus distance from the soma (radius, μm). ***E***, Using HPLC, nucleotide concentrations in the supernatants of WT and KO primary hippocampal neurons were measured. Quantification of the (***F***) cell body area, (***G***) number of primary dendrites, (***H***) number of endings, and (***I***) mean dendritic length. **p < 0.005. ****p < 0.0001. ns, non-significant. Data presented as Mean±SEM.

**Figure 3.**
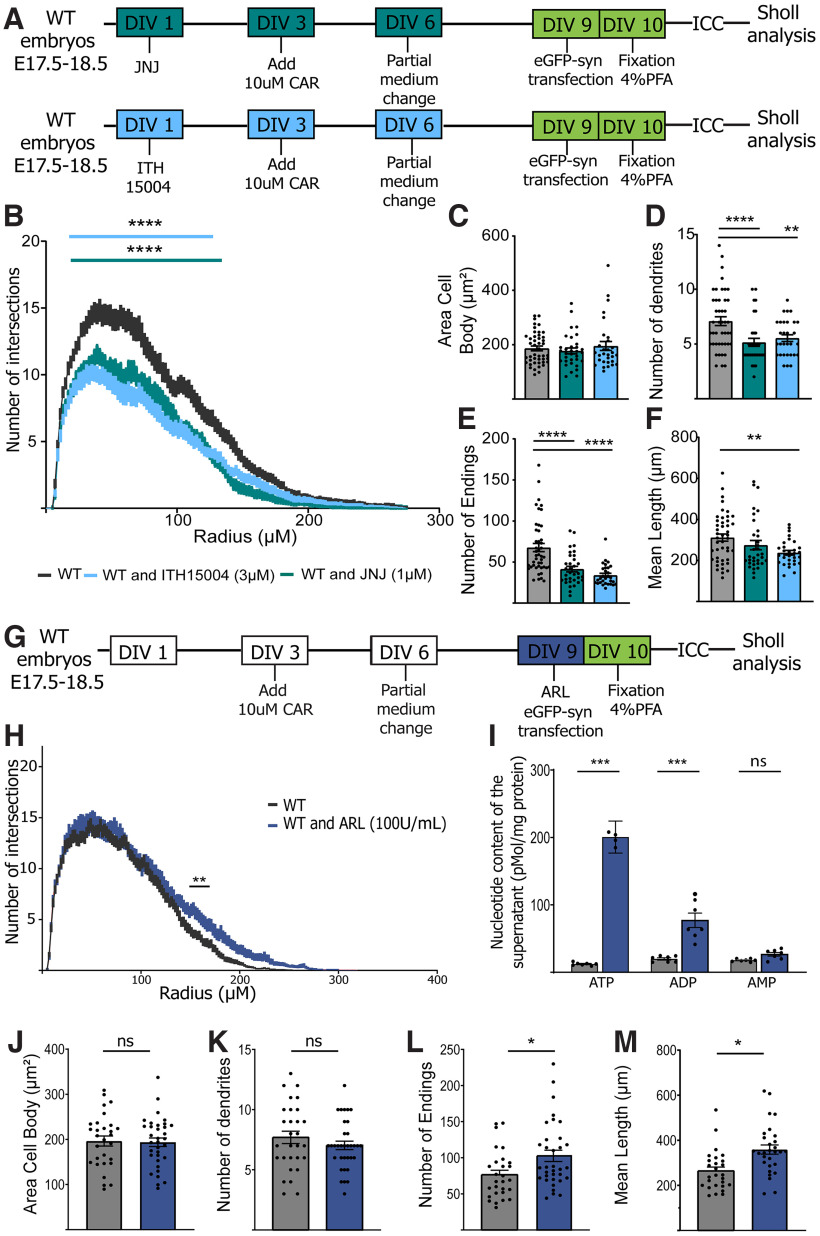
Fine-tuning effect of P2X7R on dendritic outgrowth. Antagonists disrupt normal dendritic outgrowth *in vitro*. ***A***, Scheme of the *in vitro* experiment. ***B***, Sholl analysis showing the number of intersections versus distance from the soma. Quantification of the (***C***) cell body area, (***D***) number of primary dendrites, (***E***) number of endings, and (***F***) mean dendritic length. Neurons acutely treated with ARL 67156 (100 U/ml) present elongation of dendritic endings. ***G***, Scheme of the *in vitro* experiment. ***H***, Sholl analysis showing the number of intersections versus distance from the soma. ***I***, Using HPLC, nucleotide concentrations in the supernatants of WT and ARL 67156-treated WT primary hippocampal neurons were measured. Quantification of the (***J***) cell body area, (***K***) number of primary dendrites, (***L***) number of endings, and (***M***) mean total dendritic length. *p < 0.05. **p < 0.005. ***p < 0.0005. ****p < 0.0001. ns, non-significant. Data presented as Mean±SEM.

Neurons from WT embryos were analyzed and compared with those from KO embryos (Fig. 2*A*) in addition to those isolated from WT embryos and treated with the P2X7R-specific antagonists JNJ47965567 and ITH15004 (Calzaferri et al., 2021) (Fig. 3*A*). While *P2rx7* was absent in neurons from *P2rx7* KO mice throughout development, P2X7R was only blocked in these cells after exposure to the antagonist, which was performed 4 h after plating. Again, to avoid the indirect effect of astroglia on neurons and therefore specifically assess the effect of P2X7R on neuronal growth, we eliminated astroglia by adding CAR (10 μm) at DIV3. The population of astroglia was considerably decreased under these conditions (Fig. 2*B*). Transfecting cells with a plasmid encoding GFP at DIV9 allowed us to study the morphology of individual neurons. As a control for GFP transfection, we stained transfected primary neurons for MAP2, a neuron-specific cytoskeletal protein marker that specifically stains dendrites (Fig. 2*B*). Neurons were able to be traced because of the capability of soluble GFP to fill the whole neuron with green fluorescence (Fig. 2*C*). Sholl analysis showed that KO neurons present less dendritic outgrowth (i.e., had fewer branched neurons) than WT neurons (Fig. 2*D*). HPLC analyses revealed that adenine nucleotides (ATP, ADP, AMP) were present in the medium of mouse hippocampal neurons, and we found differences in the levels of ATP and other metabolites in the supernatant between neurons from mice of the two genotypes (Fig. 2*E*). The cell body area, numbers of dendrites and dendritic endings, and mean length of each dendrite were also determined with Neurolucida software (Fig. 2*F–I*). Analysis of the number of dendrites and the number of dendritic endings (i.e., endings of any bifurcations on neurons) confirmed that KO neurons were less complex and developed than WT neurons (Fig. 2*G*,*H*). Furthermore, the decrease in the length of KO neuron dendrites revealed by Sholl analysis (Fig. 2*D*) was confirmed by analysis of the mean length of all dendrites (Fig. 2*I*).

WT neurons treated with the selective P2X7R antagonist JNJ47965567 (1 μm) presented slightly fewer dendrites than untreated WT neurons, especially near the soma (Fig. 3*B*), suggesting that treatment with the antagonist slowed the development of WT neurons. In addition to studying the impact of the widely used selective P2X7R antagonist JNJ47965567, we examined the effect of a recently developed P2X7R antagonist with a distinct chemical structure (ITH15004, 3 μm) and found that remarkably, it showed the same detrimental effect on neuronal development (Fig. 3*B*). Overall, under all conditions (neurons from WT mice, neurons from KO mice, and neurons with pharmacological blockade of P2X7R), neurons showed a typical morphology, although a reduced number of intersections was observed for both P2X7R KO and antagonist-treated neurons (Fig. 3*C–F*), which might indicate reduced dendritic growth and a less developed dendritic tree.

As an alternative approach to study the regulation of dendritic outgrowth mediated by endogenous purinergic signaling under physiological conditions, we manipulated the extracellular ATP level by acute treatment with ARL 67156 (Fig. 3*G*), a specific inhibitor of NTPDase responsible for the metabolism of ATP, 24 h before fixation (Lévesque et al., 2007). Acute ARL 67156 treatment slightly altered the morphology of primary hippocampal neurons, increasing the complexity of the neurons themselves and the process of maturation (Fig. 3*H*). A significant increase in ATP levels was observed in the medium when cells were acutely treated with the NTPDase inhibitor (100 U/ml on DIV9). This treatment also significantly increased ADP and AMP levels in the samples (Fig. 3*I*). Dendritic complexity was studied in detail by Sholl analysis (Fig. 3*J–M*), which revealed increases in the number of dendritic endings (Fig. 3*L*) and the mean dendritic length (Fig. 3*M*). This finding indicates that both ATP and its metabolites influence maturation and dendritic outgrowth and that, like P2X7R, other purinergic receptors might also participate in this regulation.

### P2X7R is need for a normal cognitive performance

**Figure 4.**
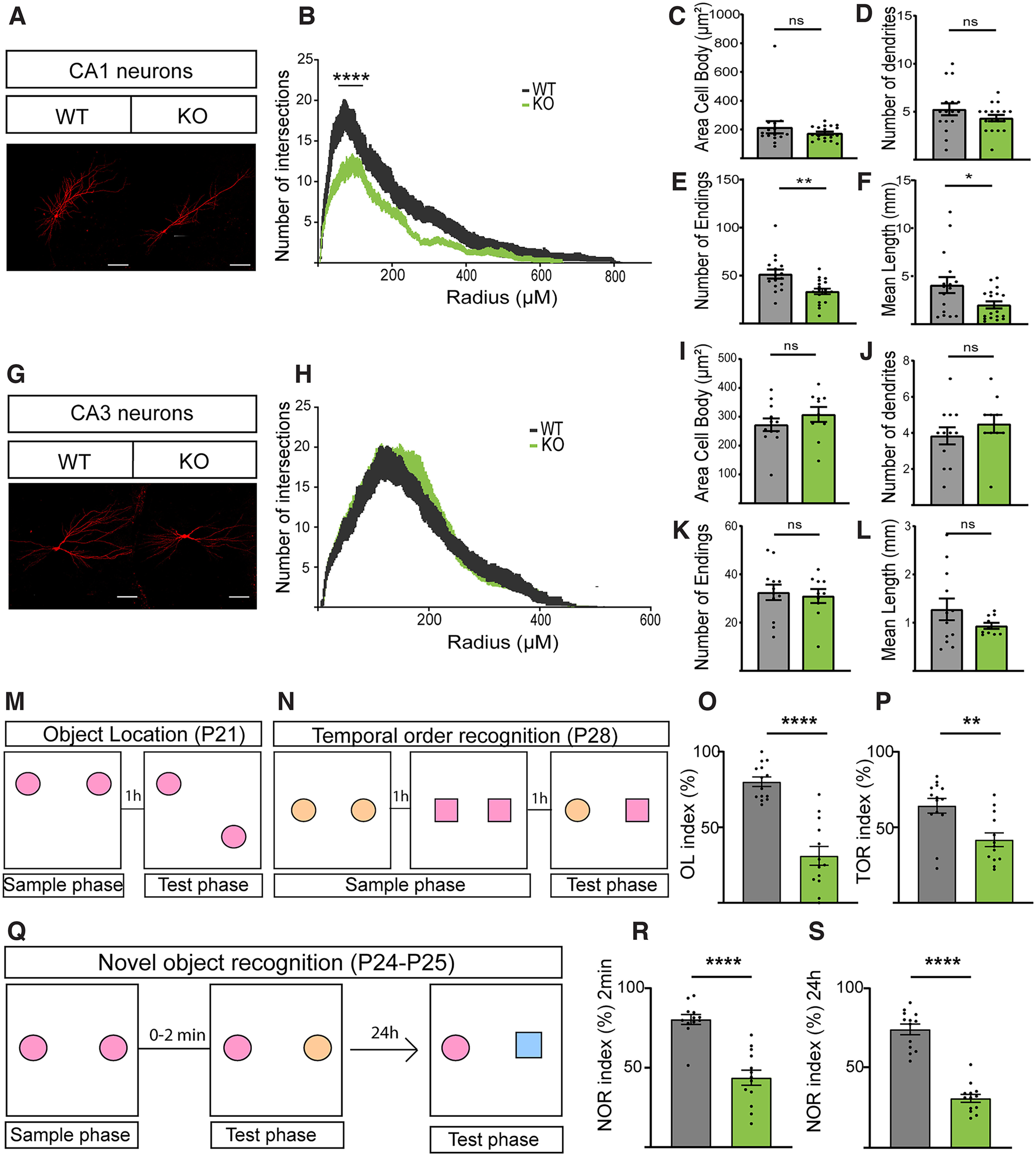
Pyramidal neurons from *P2rx7*^−/−^ mice present less dendritic outgrowth in the CA1 region of the hippocampus. ***A***, Representative pyramidal hippocampal neurons in slices of the hippocampal CA1 region from P20-29 WT and P2X7R KO mice. ***B***, Sholl analysis showing the number of intersections versus distance from the soma for WT and KO pyramidal neurons. Quantification of the (***C***) cell body area, (***D***) number of primary dendrites, (***E***) number of endings, and (***F***) mean dendritic length. ***G***, Representative pyramidal hippocampal neurons in slices of the hippocampal CA3 region from P20-29 WT and KO mice. ***H***, Sholl analysis showing the number of intersections versus distance from the soma for WT and KO pyramidal neurons. Data are mean ± SEM and analyzed using two-way ANOVA followed by Bonferroni's multiple comparison test. Quantification of the (***I***) cell body area, (***J***) number of primary dendrites, (***K***) number of endings, and (***L***) mean dendritic length. Morphologic deficits correlated with observed deficits in cognitive performance in younger animals. Different aspects of episodic memory, such as (***M***) OL and (***N***) TOR, were analyzed. Deficits in cognitive performance were observed according to both (***O***) the OL index (%) and (***P***) the TOR index (%). ***Q***, Scheme of the experiment. NOR was analyzed (***R***) 2 min after the exploration phase and (***S***) after a longer delay of 24 h. **p < 0.005. ****p < 0.0001. *p < 0.05. ns, non-significant. Data presented as Mean±SEM.

To confirm these results, pyramidal neurons in the CA1 (Fig. 4*A–F*) and CA3 (Fig. 4*G–L*) regions in acute hippocampal slices from P20-29 mice of both genotypes were studied using a method for exploring hippocampal neuronal architecture and the trisynaptic network *in vivo*. This time point represents a more advanced stage of neuronal development; however, there were apparent morphologic differences in CA1 neurons between genotypes (Fig. 4*A*), which were confirmed by Sholl analysis (Fig. 4*B*). Interestingly, these differences were not found in CA3 pyramidal neurons (Fig. 4*G–L*). The number of ramifications, which was measured as the number of endings, was significantly reduced in the CA1 region (Fig. 4*E*). We conclude that P2X7R depletion led to abnormal dendritic arborization of pyramidal hippocampal neurons in a region-specific manner, demonstrating that P2X7R is necessary for normal dendritic outgrowth in the hippocampal CA1 region but not the CA3 region during neuronal development, proliferation, and maturation.

To study the consequences of the morphologic deficits in hippocampal pyramidal neurons, mice were subjected to diverse tasks assessing cognitive functions related to the hippocampus and connections between this structure and the perirhinal and prefrontal cortices (i.e., the what, where, and when components of episodic memory) in WT and KO mice according to the protocols described by Cruz-Sanchez et al. (2020). We observed that KO mice exhibited cognitive deficits in the OL, NOR, and TOR tests, indicating that P2X7R-deficient mice might undergo a delayed neuronal maturation process (Fig. 4*M–S*).

**Figure 5.**
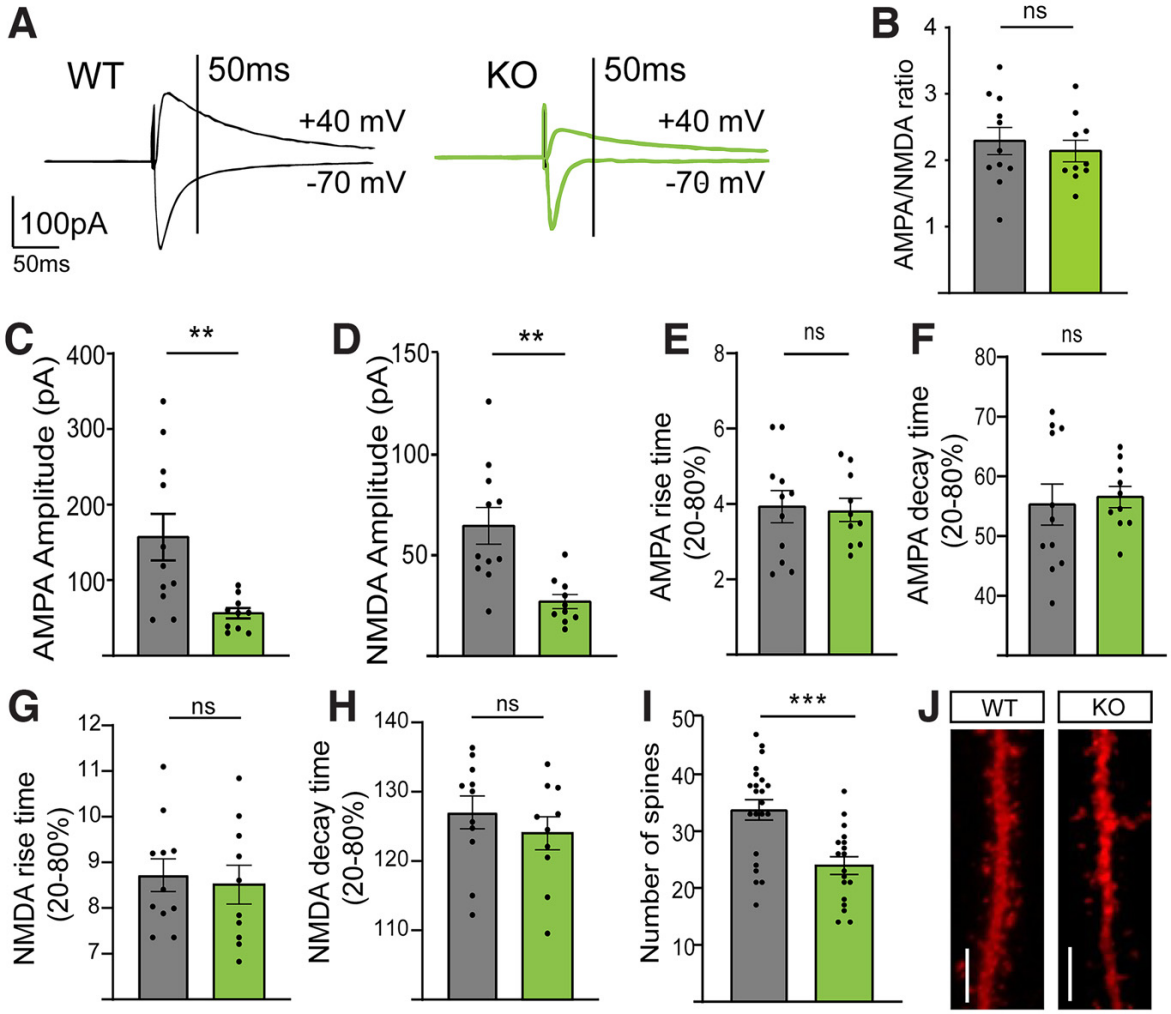
P2X7R-deficient mice present weaker synaptic strength. ***A***, Representative traces from the WT and KO groups in the presence of gabazine. ***B***, AMPA/NMDA ratio. ***C***, AMPA-mediated current amplitude. ***D***, NMDA-mediated current amplitude. ***E***, AMPA-mediated current rise time. ***F***, AMPA-mediated current decay time. ***G***, NMDA-mediated current rise time. ***H***, NMDA-mediated current decay time. ***I***, Number of spines on WT and KO neurons. ***J***, Representative segments of WT and KO neurons used to count spines. Scale bar, 50 µm. **p < 0.005. ***p < 0.0005. ns, non-significant. Data presented as Mean±SEM.

Next, we aimed to determine whether the changes in dendritic morphology change altered synaptic strength; to this end, we used a whole-cell patch-clamping technique to determine the AMPA/NMDA ratio, which is known as an indicator of synaptic efficacy. We found that the AMPA/NMDA ratio was not different between the two groups (Fig. 5*B*). However, the amplitudes of AMPA- and NMDA-mediated currents in the KO group were smaller than those in the WT group (Fig. 5*A*,*C*,*D*). Considering that the decay time constant was similar between genotypes (Fig. 5*E–H*) but that there was a marked decrease in spine number in KO mice (Fig. 5*I*,*J*), the charge was reduced in KO mice. Together, these results might indicate that KO mice display a weaker synaptic strength because of a decrease in spine density.

### P2X7R-mediated regulation of dendritic outgrowth in a neurodevelopmental model of schizophrenia

**Figure 6.**
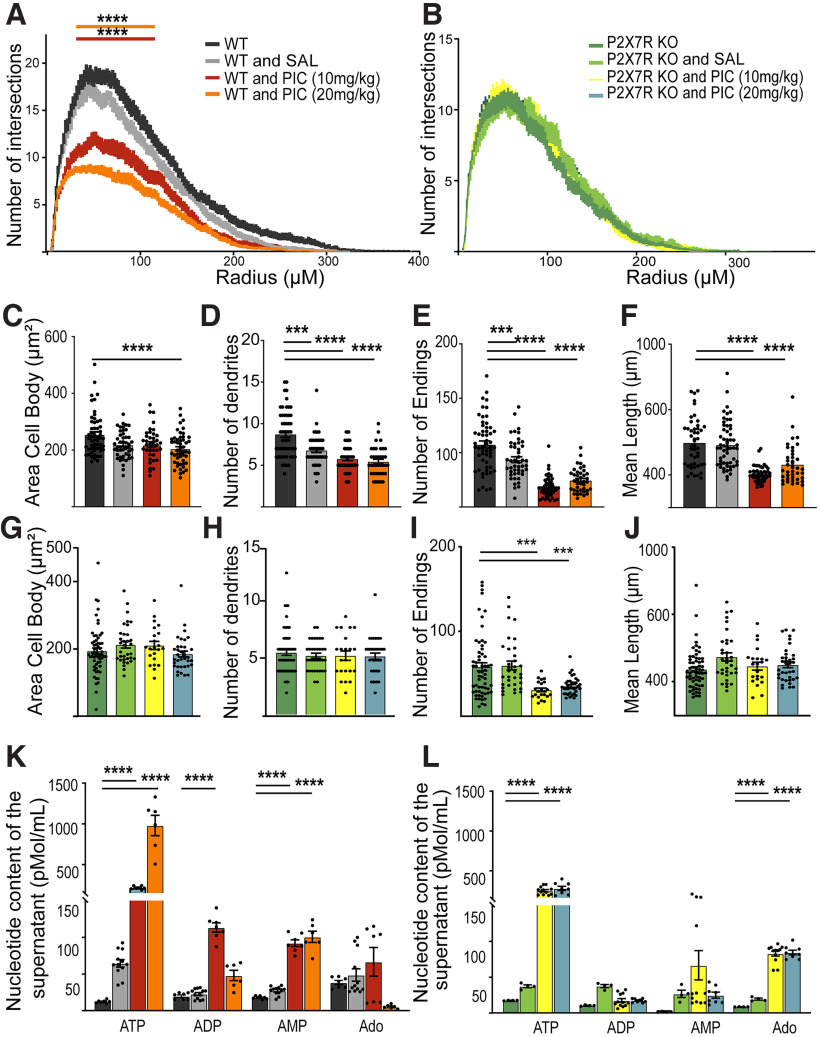
P2X7R plays a role in a neurodevelopmental model of schizophrenia. ***A***, ***B***, Sholl analysis showing the number of intersections versus distance from the soma for WT neurons, WT neurons treated with different doses of saline or PIC on E12.5, and KO neurons. Quantification of the (***C***) cell body area, (***D***) number of primary dendrites, (***E***) number of endings, and (***F***) mean dendritic length for WT neurons after different treatments. Quantification of the (***G***) cell body area, (***H***) number of primary dendrites, (***I***) number of endings, and (***J***) mean dendritic length for KO neurons after different treatments. ***K***, Using HPLC, nucleotide concentrations in the supernatants of WT and PIC-treated WT primary hippocampal neurons were measured. ***L***, Using HPLC, nucleotide concentrations in the supernatants of KO- and PIC-treated KO primary hippocampal neurons were measured. ***p < 0.0005. ****p < 0.0001. Data presented as Mean±SEM.

Morphologic alternations in dendrites could be a correlate of cognitive deficits in psychiatric disorders, such as schizophrenia and ASD. To test this concept, we constructed a model of MIA-induced schizophrenia model as described by Horváth et al. (2019) with modifications. E17.5-E18.5 control and P2X7R KO mouse embryos from pregnant mice that received intraperitoneal injection of saline or different doses of PIC (10-20 mg/kg) on E12.5 were dissected, and morphologic analysis was performed. Sholl analysis revealed dose-dependent changes in the dendritic morphology of primary hippocampal neurons from WT mice (Fig. 6*A*) but not those from KO mice (Fig. 6*B*). Interestingly, some stress-related morphologic changes (i.e., changes in the number of dendrites) (Fig. 6*D*) or number of endings (Fig. 6*E*) observed in the neurons from saline-injected animals (control group) were also observed in neurons from WT mice but not in those from KO mice (Fig. 6*B* and Fig. 6*G–J*). These findings indicate that, while P2X7R contributes to normal dendritic outgrowth of pyramidal neurons under physiological conditions, MIA compromises normal neuronal development. On the other hand, P2X7Rs seem to be necessary for development of the MIA phenotype and disruption of dendritogenesis. These observations are consistent with the schizophrenia- or autism-like phenotypes observed in young adult offspring after exposure to MIA (Horváth et al., 2019).

ATP levels were not increased in the medium of hippocampal neurons from saline-treated WT mice compared with the medium of hippocampal neurons from untreated mice. Interestingly, PIC increased ATP levels in the medium of hippocampal neurons in a dose-dependent manner (Fig. 6*K*). Significant increases in the levels of other metabolites, such as ADP, were observed after treatment with the lower dose of PIC, and the difference in the effects of the two doses was marginal. Both doses of PIC significantly increased the level of AMP in the medium (Fig. 6*K*). In neurons from KO mice, 10 mg/kg and 20 mg/kg PIC increased ATP levels in the medium to the same degree (Fig. 6*L*). ADP levels in the medium were significantly decreased after treatment with both doses of PIC. At a dose of 10 mg/kg PIC, significantly increased Ado levels in the medium, and 20 mg/kg PIC did not have a stronger effect (Fig. 6*L*). These data indicate that P2X7R mediates cell-autonomous ATP release from neurons from mice treated with high-dose PIC, resulting in a considerably higher extracellular ATP level than that in the medium of neurons derived from saline-treated or naive mouse pups. Moreover, the extracellular breakdown of nucleotides is compromised, resulting in alterations in extracellular ADP, AMP, and Ado levels. These changes are also partly mediated by P2X7R. An imbalance in extracellular purine levels might induce further changes in dendritic arborization that are distinct from those observed in the absence of pathologic signals and could lead to long-term changes in offspring behavior.

### P2X7R drives PIC-induced schizophrenic-like behavior in mice

**Figure 7.**
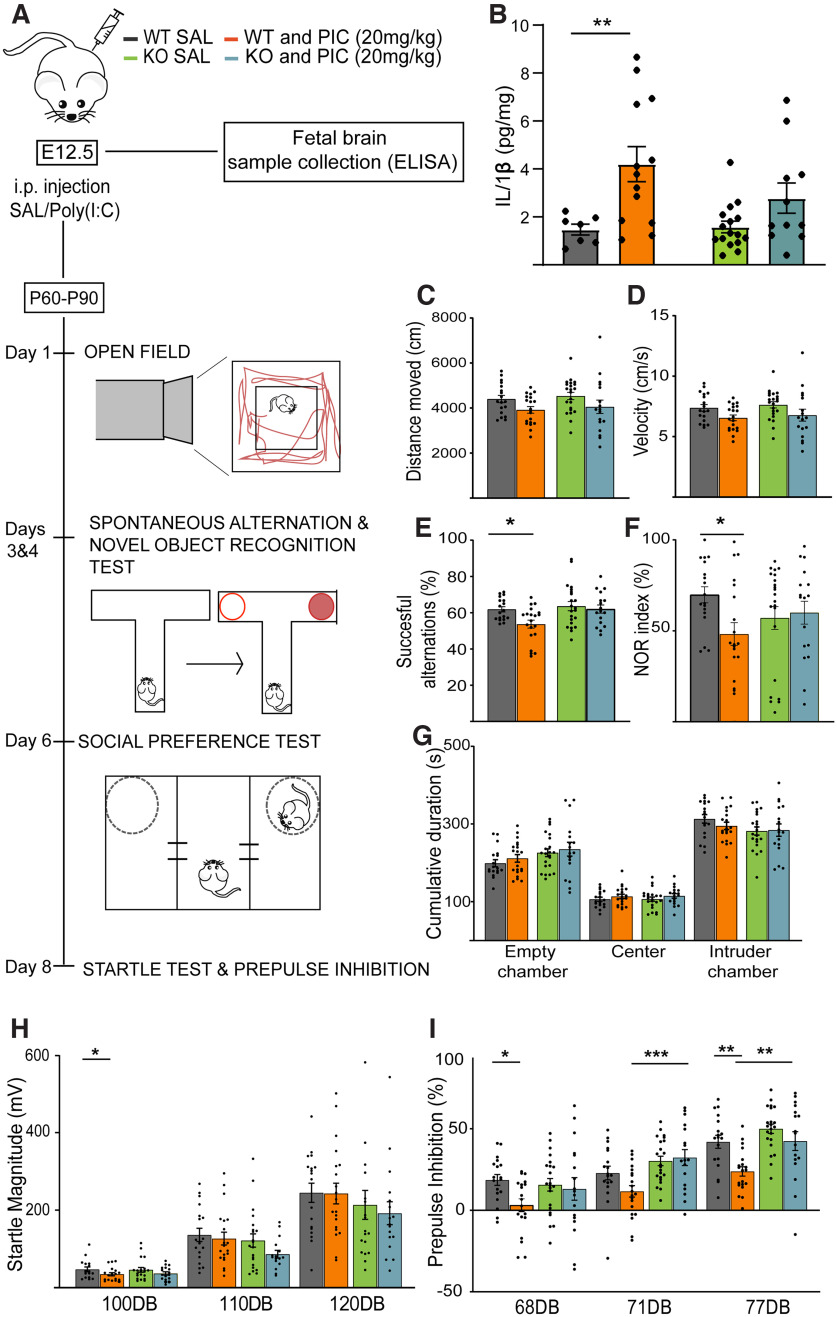
P2X7R drives PIC-induced schizophrenic-like behavior in mice. ***A***, Overview of the experimental protocol. ***B***, Maternal PIC induced elevation in the level of IL-1β in fetal brain samples of WT mice, while this effect was not observed in samples from KO. Values measured are expressed in picograms per mg of total protein. ***C***, Total distance moved (cm) and (***D***) velocity (cm/s) in the open field were not different between WT animals and PIC-treated WT animals. Quantification of (***E***) the spontaneous alternation percentage and (***F***) NOR percentage in the T-maze test showing that MIA-exposed WT animals exhibited cognitive deficits. ***G***, The amounts of time spent exploring the different cages (seconds) in the social preference test were compared between animals of different genotypes and animal subjected to different treatments. ***H***, The startle response was not different between WT and PIC-treated WT animals. ***I***, PPI was disrupted in PIC-treated WT animals. *p < 0.05. **p < 0.005. ***p < 0.0005. Data presented as Mean±SEM.

Next, we examined whether MIA elicits a schizophrenic-like behavioral phenotype in the absence and presence of P2X7R. Additionally, inflammatory changes in IL-1β levels were measured in the MIA model with the higher dose of PIC 24 h later to the injection (Fig. 7*A*). PIC induced elevation in the level of IL-1β in fetal tissue samples of WT mice, while genetic deletion of P2X7s prevented significant IL-1β elevation by PIC (Fig. 7*B*).

Then, positive symptoms and sensorimotor functions (open field exploration, Fig. 7*C*,*D*; acoustic startle reflex and PPI, Fig. 7*H*,*I*) and different cognitive functions (spatial working memory, NOR, Fig. 7*E*,*F*) were tested in female and male young adult WT and KO mice. In addition, a negative symptom (i.e., reduced sociability) was tested with the three-chamber social preference test (Fig. 7*G*). Between P60 and P90, the behavioral phenotypes of the offspring of WT and KO mice treated with 20 mg/kg PIC on E12.5 were examined (Fig. 7*A*). Interestingly, working memory in the T-maze and NOR tests, which are widely used to evaluate cognition, particularly recognition memory, was impaired in PIC-treated WT animals, but no difference was observed in the PIC-treated KO mice (Fig. 7*E*,*F*). On the other hand, MIA did not induce significant changes in behavior in the three-chamber sociability test in mice of either genotype (Fig. 7*G*). Sensory gating deficits, or the lack of ability to inhibit the response to a test stimulus, a phenomenon termed PPI, is used as an objective and relatively specific clinical biomarker of schizophrenia that can be measured in animals; therefore, it has high translational value. Here, to test the PPI, the startle reflex was measured after a loud acoustic stimulus was presented before delivery of the main pulse (120 dB). PPI is defined as reduction in the response to a stimulus. Impairment of PPI was observed in WT animals treated with 20 mg/kg PIC, but this impairment was not observed in KO animals given the same treatment (Fig. 7*H*,*I*). However, other positive symptom as hyperlocomotion was not altered (Fig. 7*C*,*D*). To summarize, we report here that MIA recapitulated some behavioral aspects of schizophrenia (i.e., PPI deficits and impairment of working memory and NOR) in WT mice treated with 20 mg/kg PIC, while negative symptoms (social deficits) were not detected in this study. Therefore, endogenous activation of P2X7R seems to be necessary for the development of particular MIA-induced positive (PPI deficits) and cognitive (cognitive deficits in the T-maze and NOR tests) schizophrenic symptoms in both male and female offspring.

## Discussion

To our knowledge, this paper is the first to report changes in dendritic outgrowth in primary hippocampal neurons from P2X7R-deficient mice. P2X7R is well known to exert pleiotropic effects as a gateway of various neuronal pathologies and neuroinflammation. Therefore, it is currently considered a therapeutic target for CNS diseases in adults and during aging (Teresa Miras-Portugal et al., 2017; Andrejew et al., 2020). Although it is expressed in the embryonic brain early on E14 (Cheung et al., 2007) and is involved in neuronal growth and differentiation regulation (Ribeiro et al., 2019), its role in early neuronal development is still unclear. Some *in vitro* studies in primary hippocampal neurons have pointed out that P2X7R has similar regulatory effects on axonal growth (Díaz-Hernandez et al., 2008). These findings might shed some light on the receptor's time-dependent expression *in vitro* and its role as a modulator of dendritic outgrowth in hippocampal pyramidal cells under physiological conditions.

The expression of P2X7R in hippocampal astrocytes and P2X7R stimulation was shown to indirectly influence the functionality of neurons (Huang et al., 2021). To exclude the contribution of astroglia, we treated our cells with an inhibitor of astroglia proliferation, which decreased the population of astroglia to ∼<10%. However, we still cannot rule out the indirect effect of signaling molecules derived from residual astrocytes on neuronal morphology, mainly when the expression of the receptor fluctuated after CAR application.

P2X7R expression in neural stem cells and immature neurons during the embryonic period was recently reviewed (Fumagalli et al., 2017). It has been demonstrated that the receptor is upregulated during embryonic stem cell proliferation and that its activity is suppressed during neural differentiation, showing that it plays a specific role in the initiation of proliferation and differentiation in earlier stages (Glaser et al., 2014). The decrease in P2X7R expression during development in our *in vitro* culture systems explains the low expression of this receptor in adulthood. These data indicate that P2X7R may initially mediate neuronal maturation and then be switched off or internalized, allowing it to respond to stimuli under pathologically high extracellular ATP levels in adulthood.

When ARL 67156, a specific inhibitor of NTPDase, was applied, extracellular ATP and ADP levels were elevated. In line with previous results, this treatment elicited a stimulatory effect on dendritic outgrowth, increasing the number of endings. This finding suggests that in our culture systems, ATP, ADP, and their breakdown products in the medium regulated the growth of dendrites through other non-P2X7-Rs, counterbalancing the action of endogenous ATP on P2X7Rs. Indeed, ATP regulates the proliferation and migration of embryonic rat-derived neural stem and precursor cells by activating various purinergic receptors in addition to P2X7R (Oliveira et al., 2015).

P2X7R-positive neurons were found in hippocampal pyramidal cell layers and other areas in the brain using isotopic ISH for P2X7R (Yu et al., 2008), in a humanized mouse P2rx7 knock-in model (Metzger et al., 2017) and recently in human excitatory but not inhibitory neurons by single-nucleus RNA sequencing (Hodge et al., 2019). In hippocampal slices, functional expression of P2X7Rs has also been demonstrated (Sperlágh et al., 2002). Additionally, we found that P2X7R deficiency affected the morphology of pyramidal neurons in the CA1 region but not in the CA3 region. This difference might suggest that this receptor has diverse effects on different cell types and in different brain areas, but this needs further investigation. Regarding the biological correlates of these results, behavioral analysis elucidated that the morphologic changes were associated with deficits in aspects of recognition and episodic memory related to different brain regions, such as the perirhinal and prefrontal cortices, but especially the hippocampus. Behavioral deficits in the three tests (i.e., the OL, NOR, and TOR tests) corresponded to deficits observed in pyramidal neurons in slices. Interestingly, these deficits in cognitive performance were not observed in adults, and no significant differences in NOR were observed in young adults. It is possible that task emergence, which is dependent on the maturation of associated brain regions, is delayed in P2X7R-deficient mice.

We also showed, for the first time, that the dendritic outgrowth is compromised in a model of MIA-induced schizophrenia in a manner mediated by P2X7R. Dendritic deficits and subsequent impairment of cognitive function in young adult offspring in this model could correlate with pathophysiological alterations in humans. Indeed, deficits in primary basilar dendrites in layers II and V of the mPFC are observed in schizophrenic patients (Broadbelt et al., 2002). Here, we report a correlation between morphologic and behavioral hallmarks in a model of neurodevelopmental disorder. These deficits were not observed in P2X7R-deficient mice, indicating a dual and opposing role for P2X7R during normal neuronal development and in neurodevelopmental psychiatric disorders. In our experiments, the endogenous activation of the receptor seemed necessary to transduce MIA leads to the development of a schizophrenic phenotype in offspring since PIC treatment did not decrease dendritic outgrowth or induce behavioral symptoms in mice with genetic P2X7Rs deficiency. Notably, deficits in the dendritic outgrowth of neurons derived from naive or saline-treated KO mice were observed in these experiments, but maternal PIC treatment did not elicit further deficits. Consistent with our data, a similar impairment of dendritic outgrowth involving the TLR3 receptor, a receptor upstream of the P2X7-NLRP3 inflammatory pathway, was also observed after prenatal PIC (Chen et al., 2017). Nevertheless, the exact mechanism whereby P2X7R activation during MIA leads to deficits in dendritic morphology and subsequent behavioral alterations requires further investigation.

Neurodevelopmental disruption in the early stages of pregnancy influences an individual's susceptibility to psychiatric disorders in later stages of postnatal life (Bayer et al., 1999), but the time window between the insult and its consequence might depend on events during development. PIC has a sex-dependent effect on the gene expression and protein levels of proinflammatory cytokines in the hippocampus when administered intracerebroventricularly (Posillico et al., 2021), which highlights the importance of the use of both sexes in the study of neuroinflammatory diseases. However, these results do not translate into the behavioral alterations observed in our MIA model. Interestingly, in an MIA model, PIC treatment was found to induce a sex-dependent decrease in the number of Purkinje cells in the cerebellum in adolescence and social deficits were observed specifically in adolescent males (Haida et al., 2019); however, such deficits were not observed in our young adult animals. Factors, such as the batch and source of PIC (Kowash et al., 2019), the caging system (Mueller et al., 2018), or even the time point of the study, can lead to variability across different studies, making it harder to accurately determine the outcomes of PIC treatment. Researchers have argued that high variability when modeling psychiatric disorders could be related to sex differences (Kokras and Dalla, 2014), but, generally, only male rodents are used. Recently, more studies have focused on the effect of MIA in females (Su et al., 2022). Here, we showed no significant differences between the two sexes (Table 2). Environmental factors can also be important in the study of neurodevelopmental diseases. Therefore, a priming/multihit model using an inflammatory reaction as a primer susceptibility factor followed by a second stressor during the juvenile period should be used in the future to increase the validity of studies of multifactorial disorders, such as schizophrenia and ASD. Despite the high variability among the treated animals, there were significant alterations in their behavior analogous to positive (PPI alterations) and cognitive (working memory deficits) symptoms of schizophrenia. The morphologic deficits in hippocampal pyramidal neurons *in vitro* corresponded with the cognitive impairments in PIC-treated WT animals but not PIC-treated KO animals.

In conclusion, our results show that higher transient expression of P2X7R is necessary for normal dendritic outgrowth during neuronal development, proliferation, and maturation. Downregulation of P2X7R after the first stages of development relegates the receptor to function under pathologic conditions in adulthood. We also showed that overactivation of the receptor *in vitro* compromises dendritic outgrowth and might contribute to developmental and cognitive deficits in an experimental model of schizophrenia. Moreover, we observed that MIA compromises normal neuronal development and influences the behavior of WT animals but not KO animals. Furthermore, endogenous activation of P2X7R seems to contribute to specific schizophrenia-like behaviors. Therefore, P2X7R has different regulatory functions depending on the stage of maturation (development or young adulthood) and whether receptor activation occurs. Additionally, activated P2X7R appears to have a pivotal role during pathologic inflammatory events in immune activation models, such as the MIA model, driving schizophrenia-like behaviors.
